# The effect of the local economic context and local public services on financial satisfaction among youth in European cities

**DOI:** 10.3389/fsoc.2024.1207807

**Published:** 2024-03-08

**Authors:** Márton Medgyesi, Ábel Csathó

**Affiliations:** ^1^TARKI Social Research Institute, Budapest, Hungary; ^2^Child Opportunities Research Group, HUN-REN Centre for Social Sciences, Budapest, Hungary

**Keywords:** subjective well-being, financial satisfaction, youth vulnerability, European cities, contextual effects, unemployment, housing, local public services

## Abstract

The post-2008 economic recovery period has seen varying degrees of improvement in the well-being of young individuals across different countries, regions, and cities of the EU. This study contributes to the literature on the geography of well-being by examining the impact of urban economic contexts on the subjective well-being of youth in Europe, a topic that has received limited attention so far. Specifically, we investigate how the local economic context has affected financial satisfaction among the young (15–35 age group) in European cities during the recovery period after the economic crisis. We study whether living in a city with better opportunities in the labor market, on the housing market, or with better local services (e.g., education or health care) affect financial satisfaction among the young. We carried out multilevel analysis of financial satisfaction among young adults on data from the Quality of Life in European Cities survey (years 2012, 2015, 2019), which asks about aspects of quality of life among a representative sample of the population in a large number of cities in EU Member States. Overall, the results suggest that a better labor market context (where it is in general easier to find a job) has a statistically significant positive effect on financial satisfaction among the young. Our results also show that satisfaction with the financial situation among young adults is significantly higher in cities with a higher quality of local social services. On the other hand, we have found only small (and statistically non-significant) contextual effect related to the local housing market.

## 1 Introduction

Youth were hit hard by the financial crisis in 2008, especially in countries more heavily affected by the economic downturn, such as those in Southern Europe (O’Reilly et al., 2015; Scandurra et al., 2021). The situation of the young in the labor market improved during the recovery period between 2012 and 2019 (Medgyesi, 2018; Eurofound, 2021), but despite these improvements the young tend to be in a more vulnerable situation on the labor market compared to other age groups. Youth unemployment tends to be higher than average (Eurofound, 2021) and when employed, the young often end up in precarious jobs given that they tend to lack experience and are still looking for the job that is the best match for their portfolio of skills (O’Reilly et al., 2015). The young face difficulties also on the housing market, as the lack of own savings makes the acquisition or rental of housing difficult particularly in housing systems where an affordable rental sector or social housing is absent (Eurofound, 2023; Kährik and Pastak, 2023).

Although studies generally find that – all else equal – well-being tends to be higher among the young than the middle-aged (Blanchflower, 2021) vulnerability on the labor market and the housing market is a threat to well-being among the young. Considerable research demonstrates the negative consequences of unemployment and job insecurity among youth on long-term labor market prospects, well-being and health (eg. De Witte, 2005; Bell and Blanchflower, 2011; Voßemer et al., 2018). Research has also demonstrated that a larger burden of housing costs also has a negative effect on subjective well-being (eg. Acolin and Reina, 2022).

In this study we analyze contextual determinants of subjective well-being among the young living in cities in Europe, which has not been explored previously in the literature. The literature on the geography of well-being has provided evidence that additionally to individual attributes (such as age, education, unemployment etc.) characteristics of the place where people live also affect well-being. Earlier literature studying contextual effects on well-being (for reviews see eg. Clark, 2018; Ballas, 2020) however focused mostly on the role of the country or regional level context. Eg. studies like Pittau et al. (2010), Aslam and Corrado (2012) or Ballas and Thanis (2022) demonstrated the significance of the regional level for well-being. The effect of the metropolitan context has been rarely explored so far in this literature. Although there are a few studies exploring the role of city-level context (see eg. Węziak-Białowolska, 2016; Gajdoš and Hudec, 2020), to our knowledge no studies have explored the effect of the metropolitan context for subjective well-being among the young. Studies which consider contextual determinants of well-being among the young also tend to focus on the country-level context, eg. analyzing the impact of employment protection legislation and labor market policies on well-being among the young (eg. Russell et al., 2020). Analyzing the effects of city-level context on subjective well-being specifically for the young is relevant given that the young constitute a vulnerable group on the labor market and the housing market and the effects of the social context on well-being can be different across social groups (Ballas, 2013).

This study aims to contribute to the literature by filling this research gap. It investigates how subjective well-being of the young in European cities is affected by aspects of the metropolitan context, focusing on the effects of the urban labor and housing market and local public services. Cities have been shown to be engines of economic prosperity, being characterized by high levels of productivity, innovativeness, and entrepreneurship. At the same time economic and population growth has been uneven among metropolitan areas of the EU during the past decades. Most of the demographic and economic growth has been concentrated in the large cities of the core Western European area, while population and economic activity declined in weak market cities which have lost their central position during the transition to the post-industrial era (d’Ovidio and Ranci, 2014; Eurostat, 2016). The urban economic literature highlights that attractiveness of cities also depends on location-specific attributes such as the local environment, public goods and services and local government policies (Lambiri et al., 2007). But there is a downside to living in successful cities. Higher labor productivity (and resulting higher wages) and higher quality of local services and other city amenities tend to be reflected in higher housing prices (Florida et al., 2020; Glaeser, 2020).

In our study we expect that such differences in urban economic environment and in the quality of local public services are also important for well-being of the young. The characteristics of the local labor market, the local housing market and the quality of local public services determine the opportunities young adults have and the constraints they face in relation to critical areas of well-being. In our study we investigate the effect of the urban economic context and public services provided by the cities on one subdomain of subjective well-being, namely financial satisfaction. Does having a well-functioning local labor market and local housing market in the city contribute to financial satisfaction among the young? Do local public services contribute to increasing financial satisfaction among the young in the urban environment? To answer these questions, we carried out a multilevel regression analysis on data from the Quality of Life in European Cities survey, using waves between 2012 and 2019.

In the following analysis we first outline the hypotheses of our research based on a review of the literature (section 2), we present the data and the methodology used (section 3), which is followed by the presentation of the results of the analysis (section 4) and in the final section we provide a discussion of the findings.

## 2 Literature review and hypotheses

### 2.1 Approaches to the notion of financial satisfaction

As described above, in our study, we use financial satisfaction as the main outcome variable. Financial satisfaction is discussed in the literature in relation with the concepts of financial well-being and life satisfaction. Brüggen et al. (2017) define financial well-being “as the perception of being able to sustain current and anticipated desired living standards and financial freedom” (Brüggen et al., 2017:229). According to this approach, financial satisfaction is a subjective indicator of financial well-being, which focuses on satisfaction with the current situation of the household. Although financial satisfaction does not reflect expectations about the ability to finance the desired life in the future (Brüggen et al., 2017) it is widely used as a measure of financial well-being in the literature.

In the literature about life satisfaction, financial satisfaction is usually seen as one of the domains of general life satisfaction besides other factors, such as satisfaction with personal relations, job, housing or health (Van Praag et al., 2003; Van Praag and Ferrer-i-Carbonell, 2008; Medgyesi and Zólyomi, 2016). The literature demonstrates, that the association between financial satisfaction and life satisfaction is important. In the study of Van Praag et al. (2003) it is the domain having the strongest link with overall life satisfaction. Medgyesi and Zólyomi (2016) found rank correlation coefficient R = 0.6 between financial satisfaction and life satisfaction in European countries using EU-SILC data^1^, while Ngamaba et al. (2020) report an average correlation of R = 0.4 in their meta-analysis of 24 studies.

The following section summarizes relevant results from studies analyzing financial satisfaction. Nevertheless, as financial satisfaction tends to be strongly associated with overall life satisfaction, studies about general life satisfaction can also provide important insights for formulating our expectations.

### 2.2 The impact of the local context on financial satisfaction among the young

The social science literature has extensively studied how the subjective well-being of individuals is affected by characteristics of the macro-level context (city, county, region, or country) of their place of living (Ballas and Tranmer, 2012; Ballas, 2013; Węziak-Białowolska, 2016; Hastings, 2019). The contextual variables covered by these studies include characteristics related to the economic context (e.g., the level of economic development and unemployment), contextual inequality, indicators of urbanization, and the characteristics of local services and policies.

For example, regarding the effect of economic development, Easterlin, in his groundbreaking work, pointed out that there is no unequivocal relationship between happiness and economic development. According to his results, the positive effect of income on happiness that is found within and between countries in cross-sectional data does not apply for developments over longer time periods (Easterlin, 1995). Although subjective well-being changes with the short-term fluctuation of business cycles (Stevenson and Wolfers, 2008; Hovi and Laamanen, 2016) there seems to be no association between long-term changes in income and subjective well-being (Easterlin et al., 2010)^2^. Similar studies have explored the association between characteristics of the macro-level context and financial satisfaction. For example, Hastings (2019) found no significant effect of changes in state level median income and individual level financial satisfaction in the US over the period between 1973 and 2012. According to the author this is consistent with relative income being more important for financial satisfaction compared to absolute income.

Studies also discuss through what channels economic development might affect financial satisfaction. For example, a higher GDP/capita in a city is likely to be associated with a better labor market, resulting in higher wages and/or lower levels of unemployment. It might also be that more affluent cities can afford more accessible and better quality local public services, which might also improve well-being. On the other hand, prices – and, most importantly, housing prices – tend to be higher in more prosperous cities, which might negatively affect financial satisfaction.

Several studies in the literature demonstrate the effect of the labor market context, showing that macro-level unemployment rate has a negative impact on financial satisfaction (Kose and Cinar, 2020; Lee et al., 2023). Lee et al. (2023) examined the effect of different macroeconomic variables on financial satisfaction using data from the World Values Survey and found that the countries’ unemployment rate and the inflation rate have a negative influence on financial satisfaction. Kose and Cinar (2020) also found that the country-level unemployment rate shows a clear negative effect on financial satisfaction. These results are similar to those obtained by studies investigating the association between unemployment and life satisfaction (Di Tella et al., 2001; Lawless and Lucas, 2011). This literature found similar results in studies focusing on young adults (Russell et al., 2020) and by studies analyzing variation between cities rather than countries. Florida et al. (2013) show that a higher (and increasing) unemployment rate decreases average life satisfaction in a sample of US cities and similar results were found by Węziak-Białowolska (2016) using city-level data from EU countries.

One explanation for such result might be that a higher unemployment rate can reflect increased work insecurity, meaning a higher risk of job loss for those in employment and more competition for jobs among the unemployed (Koster, 2023). In addition to this effect on individual labor market chances, a higher unemployment rate might also be related to the adverse social consequences of increased poverty or inequality – such as higher public expenditure on social benefits and health care or lower level of social cohesion (Wilkinson and Pickett, 2010) – which might negatively impact financial satisfaction of all residents of the city. Because of these external effects, we expect that a better labor market context (where it is in general easier to find a good job or where the unemployment rate is lower) will have a positive effect on financial satisfaction among the young (Hypothesis 1).

We are also interested in analyzing the effect of the housing market context on financial satisfaction of the young. The literature argues that the effect of housing prices on individual financial satisfaction depends largely on whether one is a homeowner or lives in rented housing (Atalay and Edwards, 2022). The effect of an increase in housing prices differs by tenure status due to the dual nature of housing. On the one hand, housing can be seen as a consumption good and in increase in housing prices raises the consumption costs of housing which decreases financial satisfaction. In the same time an increase in housing prices also raises housing wealth of homeowners, which is expected to have a positive effect on financial satisfaction, canceling out the negative effect of rising consumption costs for these households (Atalay and Edwards, 2022)^3^. Among young adults homeownership is less frequent as most of them are renters or living with parents preparing to enter the housing market (Hvozdetska et al., 2022; Eurofound, 2023; Kährik and Pastak, 2023). Therefore, we can expect that – at the individual level – higher housing prices (or lower perceived housing affordability) should have a negative effect on individual financial satisfaction.

Regarding the contextual effect of housing unaffordability (high housing prices), a range of studies suggest a negative impact on financial satisfaction. According to the literature low housing affordability can lead to a range of social problems such as increased homelessness, deteriorating health (Baker et al., 2020) and might also negatively impact child outcomes in the families concerned (Holme, 2022). In addition, unaffordability of housing can also contribute to class-based residential segregation, when low income people are priced out from more attractive neighborhoods (or cities). The literature shows that socioeconomic segregation has increased not only in the US, but in several European capital cities as well (Musterd et al., 2017). Research has also documented the high social costs of class-based and racial segregation like lower tax revenues, increased costs of delivering public services, reduced government spending on productive public goods, lower competitiveness and constrained economic performance of metropolitan areas (Turner and Rawlings, 2016).

On the other hand, the literature suggests that lower housing affordability (high housing prices) may also be related to attractive aspects of a city/neighborhood (e.g., proximity to jobs, large green areas, high-quality social services, etc.), which increase the well-being of inhabitants (Ballas, 2013; Glaeser, 2020). Therefore, high housing prices might appear to be positively associated with well-being in studies where these attributes of neighborhoods are not fully controlled. Accordingly, Rentfrow et al. (2009) found that happiness is higher in states where median housing prices are higher and similar results were reported by Florida et al. (2013) and Tan and Guan (2021) at the city level. Overall, as there are arguments both for a negative and a positive effect of city-level housing affordability on financial satisfaction, our expectation is that the contextual effect in this case should be weak (weakly positive or negative) after controlling for individual perceptions of housing affordability (Hypothesis 2).

We could not find any study on the effect of local public services on financial satisfaction, therefore our study is the first to explore this relationship. The literature about the impact of such services on life satisfaction suggests that better local public services can also improve subjective well-being (Węziak-Białowolska, 2016; Gajdoš and Hudec, 2020). As here we are interested in determinants of financial satisfaction among the young, the most interesting services are those that affect the financial situation of individuals. In our analysis we included good quality health care and educational facilities, which contribute to the human capital accumulation of the young and their children. In addition, the quality of public transport is important for improving access to labor markets in more remote parts of a city. According to Moeinaddini et al. (2020), satisfaction with healthcare services and public transport is among the factors most strongly associated with city-level life satisfaction. Thus, we expect that young adults’ satisfaction with local services (health care, education, public transport) will be associated with greater financial satisfaction. In addition, the literature also argues that social services can have important positive external effects, such as lower public expenditure due to improved population health, reduced crime rates (see Grossman, 2006) or lower unemployment rates (Bastiaanssen et al., 2020, 2022). Therefore, high quality services can have positive effect on financial satisfaction not only through direct satisfaction from the use of services but also by creating public goods for the community. Overall we expect that high quality public services also have a positive contextual effect on financial satisfaction among the young (Hypothesis 3).

In our analysis we also control for other characteristics of cities that might affect financial satisfaction. For example, extensive literature studies how urbanization and city size play a role in subjective well-being. According to Hastings (2019) financial satisfaction is the highest among those living in rural areas and lowest among those living in suburbs, with urban inhabitants falling in between. Population density has been found to be negatively correlated to financial satisfaction in states of the United States (Hastings, 2019). Regarding the relation between population density and life satisfaction the literature suggests that in Western-European countries and the United States life satisfaction tends to be higher in smaller towns than in bigger cities (Bergstad et al., 2012). Okulicz-Kozaryn and Valente (2019) find interesting generational differences in this regard: while members of the older generation are happiest in small settlements and have the lowest subjective well-being in large cities, Millennials (born between 1982 and 2004) are happiest in cities with more than 50,000 inhabitants.

Other authors emphasize the role of income inequality as contextual predictor of financial satisfaction. As we mentioned earlier, several studies have stressed the importance of relative income in shaping financial satisfaction (Easterlin, 1995; Newman et al., 2008) and this implies that an increase in inequality can have a negative effect on financial satisfaction if it means declining relative income compared to one’s reference group. Hastings (2019) found that rising inequalities affected overall financial satisfaction negatively and this negative effect proved to be strongest in the middle income quintiles. Other studies found that higher city-level inequalities also result in lower levels of life satisfaction (Glaeser et al., 2009).

### 2.3 Financial satisfaction among the young: the effect of individual-level attributes

The literature identifies several demographic and socioeconomic attributes of individuals that correlate with financial satisfaction. Financial satisfaction tends to follow a U-shape in terms of age, with a negative overall tendency (Van Praag and Ferrer-i-Carbonell, 2008; Medgyesi and Zólyomi, 2016; Hastings, 2019), which is similar to the result found in research on life satisfaction. In this regard Blanchflower (2021) found a U-shaped relationship with a negative overall tendency in a wide variety of countries, both developed and developing. Regarding the effect of gender, the review of She et al. (2022) found more reports of higher financial well-being among women, as men more often use higher income as a measure of success. Despite this, several studies with opposite findings were also mentioned (She et al., 2022). Findings about gender effects are also inconsistent in studies on life satisfaction (see reviews in Proctor et al., 2009; Batz and Tay, 2018). For instance, in Węziak-Białowolska (2016) and She et al. (2022), women are found to be more satisfied with life, while in Ballas and Tranmer (2012), the opposite is claimed to be true. Nevertheless, it is important to note that the difference is relatively small in all three cases.

The role of education could be expected to be unequivocally positive on financial satisfaction since a higher-level education leads to a better job and higher income. However, with higher level of education, the reference group may also change (Newman et al., 2008), and as psychological research has pointed out, a higher-level education may lower satisfaction due to the more difficult fulfilment of aspirations (Ferrante, 2009). Accordingly, while the level of education usually shows a rather positive impact on financial satisfaction (Medgyesi and Zólyomi, 2016; Kose and Cinar, 2020), the effect is not always significant (Hastings, 2019), or not linear (Tharp et al., 2019). In fact, we can also find results, where years of education has a significant negative impact on financial satisfaction (Van Praag and Ferrer-i-Carbonell, 2008). The fulfilment of aspirations may be especially difficult in the case of young adults, who, at the beginning of their careers, may not have the opportunity to find a job that matches their qualifications (see, e.g., Khattab and Fenton, 2009).

Individual experience of unemployment has an adverse effect on financial satisfaction (see, e.g., Newman et al., 2008; Medgyesi and Zólyomi, 2016; Hastings, 2019; Kose and Cinar, 2020). These results are similar to those obtained in studies analyzing the relation between unemployment and life satisfaction. According to these findings, the young who manage to find a full-time job after leaving school are significantly more satisfied with their life than those who are neither at school nor in full-time employment (see Feather and O'Brien, 1986; Creed et al., 2003; Unt and Täht, 2020). Moreover, a long-term ‘scarring’ effect can be identified after the experience of unemployment (Baranowska-Rataj et al., 2016). Apart from being employed, the type of employment is also an important factor: having an occupation with higher prestige was found to significantly and positively affect life satisfaction (Martikainen, 2009). Stanojević et al. (2016) found that both unemployed young adults and precarious workers tend to be less satisfied with their job situation than employees.

Not surprisingly, income has also a positive impact on financial satisfaction: richer individuals report higher level of satisfaction with their financial situation. However, this impact is not necessarily linear as additional income tends to exert smaller positive effect on richer people than on poorer ones (Ferrer-i-Carbonell, 2005; Newman et al., 2008). Besides absolute income, there is also a positive impact of relative income on financial satisfaction either if we compare income to a reference group or to other household members (Newman et al., 2008). Vera-Toscano et al. (2004) add that the opportunity to save money monthly also increases financial satisfaction, though they also point out that it is rather the opportunity of saving itself, and not the amount what really matters.

## 3 Materials and methods

### 3.1 Data

The analysis uses the European Commission’s Perception Survey on the Quality of Life (QoL) in European Cities, which has been fielded six times since 2004. We used data from 2012, 2015 and 2019 as these waves of the survey included the question regarding satisfaction with financial situation (European Commission, 2012, 2015, 2019). In addition, the surveys contain questions about respondents’ opinion about the local economy (labor market, housing market) and about satisfaction with public services such as public transport, healthcare, education services. Besides the questions about satisfaction with different aspects of city life, the surveys also collected information about the respondents’ occupation, education, age, gender, household size, and how long the respondent had lived in the city.

All three waves of data used here include all current European Union member countries plus the United Kingdom, Switzerland, Norway, Iceland and Turkey, but in the final analysis sample data from Cyprus, Iceland and Romania was not used due to missing data on GDP.^4^ The study covers the countries’ capitals plus up to five other cities depending on the size of the country in each year. Our analysis sample includes 69 cities (see Table 1), but as GDP data are missing for Zürich and Geneva in 2019, the total number of city-years in the analysis is 205. The data was collected via mobile and landline telephones; discussions were in the respondent’s national language. In 2012 and 2015 500 people aged 15 or older were surveyed per city using probability sampling, while in 2019 the sample size was increased to 700 respondents per city. The selection of the sample was implemented through random digit dialing in the case of the landline sample and through national phone number registers in the case of mobile phones (European Commission, 2019). At this first stage, attention was paid to ensuring that the numbers were proportionally associated with population of the city’s different districts, where possible.

**Table 1:** Cities included in the analysis and sample size of 15–35 age group (2012–2019).

Country	City	*N*	Country	City	*N*
Austria	Graz	371	Lithuania	Vilnius	534
	Wien	382	Luxembourg	Luxembourg	399
Belgium	Antwerpen	434	Latvia	Riga	442
	Bruxelles	476	Malta	Valletta	397
	Liege	416	Netherlands	Amsterdam	443
Bulgaria	Burgas	510		Groningen	555
	Sofia	487		Rotterdam	506
Croatia	Zagreb	444	Poland	Bialystok	626
Czechia	Ostrava	407		Gdansk	590
	Praha	431		Krakow	632
Denmark	Aalborg	432		Warszawa	573
	Kobenhavn	562	Portugal	Braga	569
Estonia	Tallinn	431		Lisboa	438
Finnland	Helsinki	528	Slovakia	Bratislava	457
France	Bordeaux	475	Slovenia	Ljubljana	393
	Lille	509	Spain	Barcelona	419
	Marseille	385		Madrid	440
	Rennes	515		Malaga	469
	Strasbourg	477		Oviedo	389
	Paris	489	Sweden	Malmo	463
Germany	Berlin	470		Stockholm	424
	Rostock	280	United	Belfast	429
	Hamburg	427	Kingdom	Cardiff	475
	Leipzig	405		Glasgow	500
	Munchen	478		London	546
Greece	Athina	492		Manchester	499
Hungary	Budapest	392		Tyneside con.	428
	Miskolc	367	Norway	Oslo	545
Ireland	Dublin	494	Switzerland	Geneve	482
Italy	Bologna	344		Zurich	387
	Napoli	443	Turkey	Ankara	602
	Palermo	385		Istanbul	744
	Roma	265		Antalya	610
	Torino	383		Diyarbakir	1,063
	Verona	264			

### 3.2 Definition of variables

The impact of the local economic context and public services on financial satisfaction among the urban young (15–35 age group) was analyzed with multilevel regression models. The dependent variable of our analysis is respondents’ financial satisfaction. This item involved asking respondents: “On the whole, are you very satisfied, fairly satisfied, not very satisfied, or not at all satisfied with the financial situation of your household?.” Responses were coded on a 4-point scale, ranging from very unsatisfied to very satisfied. The distribution of the dependent variable in each city is shown in Supplementary Figure S1.

The main independent variables in our analysis are indicators of the economic context (the local labor market and housing market) and satisfaction with local public services. As a subjective indicator of the local labor market we used agreement of respondents with the following statement: “It is easy to find a good job in my city.” Responses were coded on a four-point scale, from strongly disagree to strongly agree. Our subjective indicator of the housing market context is based on agreement with the statement “It is easy to find good housing in my city at a reasonable price.” In both cases we transformed these variables into a dummy variable which takes value of 1 in case the respondent agrees or strongly agrees with the statement. These variables regarding individual opinions about the labor market and the housing market were included as independent variables in our multivariate statistical analysis. But the main variables of interest were contextual-level indicators of the local labor market and housing market, which were constructed by calculating the percentage of respondents agreeing or strongly agreeing with the statements in each city in each year.

As our indicator of quality of local public services, we use a service satisfaction index which is based on the city dwellers’ subjective satisfaction with local services. The index is based on three items: satisfaction with education services, healthcare services, and public transport. The wording of the questions in case of education services was as follows (similar questions were asked in case health care services and public transport): “Please tell me if you are very satisfied, rather satisfied, rather unsatisfied or not at all satisfied with schools and other educational facilities in your city or area.” We constructed and indicator of individual satisfaction with public services by averaging responses to these questions for every respondent. The contextual-level policy satisfaction index is defined as the mean value of individual satisfaction scores for each city for each year. The Cronbach-alpha for these three variables equals 0.8, which suggests a good internal consistency of this set of items.

Ziller and Andress (2022) argue that aggregated survey responses can represent the quality of of public services in a more reliable way than structural indicators of municipality characteristics as structural indicators tend to suffer from cross-country comparability problems. In addition, subjective indicators are also appealing for a more substantive reason, as contextual characteristics are susceptible to influence attitudes only to the extent that they are perceived. Despite this, we provide robustness analysis by using objective indicators such as the unemployment rate in case of the labor market and rental prices in case of housing affordability.

### 3.3 Empirical strategy

Multilevel models are used in the social sciences when the structure of the data is hierarchical; that is, when individuals (level-1 units) in the population are grouped into clusters (e.g., cities, school classes, countries; level-2 units). Multilevel models take into account the correlation between individuals from the same cluster. These models allow for introducing group-level explanatory variables, estimating correct standard errors for these variables, and studying cross-level interaction effects (Snijders and Bosker, 2012). As financial satisfaction is measured on an ordinal scale we used multilevel ordered probit estimation. Bryan and Jenkins (2016) note that at least thirty level-2 units are needed for a reliable estimation of contextual-level effects in a multilevel nonlinear regression model. In our sample, we have 205 level-2 units, so this requirement is satisfied.

In our regression analysis satisfaction with financial situation is explained by the main contextual (city-year level) independent variables about the local labor market, the housing market and local services in a multilevel model with city-year-level random intercepts and we also add fixed effects for years of the survey. In additional specifications we added country fixed-effects to the model so in these models the estimated effects of contextual variables reflect differences between city-years within countries rather than differences between countries.

The literature warns that to uncover a contextual effect one needs to study the effect of the level-2 variable after accounting for the corresponding level-1 variable (Leyland and Groenewegen, 2020). Therefore, the individual-level opinions about the local labor market (agreement with finding job is easy), housing market (agreement with finding affordable housing is easy) and individual satisfaction with public services were also included in the model. We suppose that these variables capture the direct effect of respondents’ own perceptions and experiences on financial satisfaction. The variables aggregated to the city-year level on the other hand capture a contextual effect (of the labor market, housing market or public services) which affects all city residents regardless of their individual-level perceptions. In addition, we control for a range of contextual and individual variables in our regression models. As contextual-level control variables, we use the population of the cities and GDP *per capita* from the OECD database of metropolitan areas (OECD, 2022), which describes functional urban areas with more than 250,000 inhabitants. Unfortunately, it proved impossible for us to find reliable data on income inequality for a sufficiently large number of the cities included in our sample. Nevertheless, we control for urban inequality of educational attainment in our robustness analysis using aggregated data from the survey used in the analysis.

Individual-level control variables were also included in the model, namely gender, age (years), education level (low /medium /high), household composition (married or cohabiting couple, no children /single parent household /married or cohabiting couple with children /other) and occupation (manager /professional /white collar /blue collar /unemployed /student /inactive). Unfortunately, the survey does not record objective information on household incomes or wealth holdings, therefore our main estimates control only for determinants of income status such as age, education or occupation. The only variable directly referring to the household’s living standards in the survey is a subjective variable, which asks respondents about having difficulties to pay bills at the end of the month within the last 12 months (most of the time / from time to time / almost never or never). We checked robustness of our results with the inclusion of this variable in the model. The distribution of independent variables included in the analysis is shown in Supplementary Table S1.

## 4 Results

**Figure 1:**
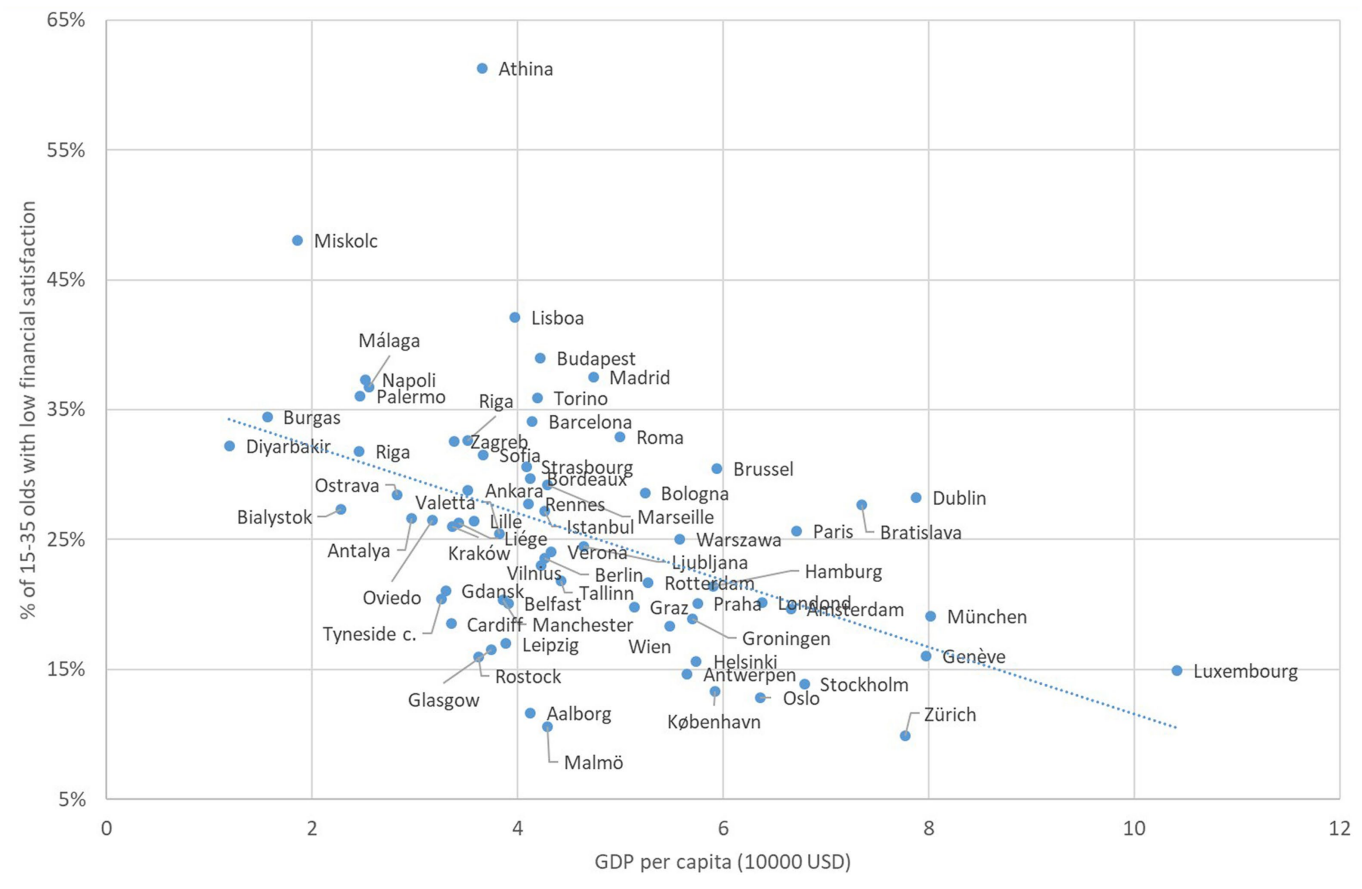
Association of GDP *per capita* and low financial satisfaction among the young, 2012–2019. Average values over the years 2012, 2015, 2019.

As an illustration of our data the following graph shows the bivariate relationship between GDP *per capita* and financial satisfaction in the cities. As Figure 1 shows there seems to be a weak negative association between GDP *per capita* and the percentage of those reporting low financial satisfaction (being not very satisfied, or not at all satisfied with their situation). During the 2012–2019 period the percentage of those with low financial satisfaction was the highest in Athens, where 61% of the young were unsatisfied with their financial situation. Cities with high levels of unsatisfied young include Miskolc (48%), Lisbon (41%), Budapest (39%) and also Madrid and Palermo (37%). Cities with the lowest percentage of young with low financial satisfaction were Zürich (10%), Malmö (11%) and Aalborg (12%).

In order to better understand the association between local economic development and financial satisfaction among the young we conducted multilevel regression analysis to control for contextual-level and individual-level confounders. In the first step of the multilevel analysis we ran a null model with only the random city-year level intercept and a second model also including individual-level control variables. These are shown in the Supplementary Table S2. Variance between the level-2 units (city-years) in case of the young is 8.4% of total variance, which slightly declines to 7.8% with the introduction of level-1 (individual-level) variables.

The effect of GDP *per capita* is also significant in a multilevel model including individual-level controls (see Supplementary Table S3). Cities with higher *per capita* GDP might offer more and better paid employment opportunities for the young and might also provide public services of better quality. On the other hand, costs of living – especially of housing – tend to be higher in these cities. The results of multilevel models including the contextual-level variables of interest and control variables are shown in Table 2^5^. Model 1 suggests that satisfaction with financial situation among the young tends to be higher in city-years where people more strongly agree that it is easy to find a job on the labor market and where people are more satisfied with public services. On the other hand, living in a city where people agree more that it is easy to find affordable housing has only a small positive effect, which is not statistically significant. If the corresponding level-1 variables are included in the model (see Model 2), the contextual effects (the effects of city-year level variables) regarding the labor market and public services remain positive and statistically significant, although the coefficients become smaller^6^. In case of the level-1 variables we see strong positive effects. The young agreeing that it is easy to find a job, agreeing it is easy to find affordable housing and who are more satisfied with public services all tend to be more satisfied with their financial situation.

**Table 2:** Effects of local economic context and local public services on satisfaction with financial situation among the young (standardized coefficients from multilevel ordered probit models).

	Model (1)	Model (2)	Model (3)	Model (4)
Agree: easy to find job (individual)		0.180^***^		0.181^***^
		(0.000)		(0.000)
Agree: easy to find job (city-year)	0.150^***^	0.090^***^	0.143^***^	0.074^**^
	(0.000)	(0.000)	(0.000)	(0.001)
Agree: easy to find housing (indiv.)		0.139^***^		0.137^***^
		(0.000)		(0.000)
Agree: easy to find housing (city-year)	0.044	−0.002	0.084^***^	0.035
	(0.109)	(0.954)	(0.000)	(0.090)
Satisfaction with services (indiv.)		0.196^***^		0.196^***^
		(0.000)		(0.000)
Satisfaction with services (city-year)	0.156^***^	0.070^***^	0.059^*^	−0.015
	(0.000)	(0.001)	(0.012)	(0.533)
Population	−0.023	−0.020	0.018	0.020
	(0.260)	(0.343)	(0.228)	(0.190)
GDP *per capita*	0.045	0.057	−0.018	−0.004
	(0.112)	(0.052)	(0.562)	(0.906)
Individual controls	Yes	Yes	Yes	Yes
Year dummies	Yes	Yes	Yes	Yes
Country dummies	No	No	Yes	Yes
Variance city-year level	0.032	0.034	0.002	0.002
*N*	28,130	28,130	28,130	28,130
Number of city-years	205	205	205	205
Log-likelihood	−31974.5	−31256.2	−31840.8	−31123.2
LR test	0.000	0.000	0.011	0.004

Standardized beta coefficients; *p* values in parentheses, ^*^p < 0.05, ^**^p < 0.01, ^***^p < 0.001.

In Models 3 and 4 we repeated the analysis with the inclusion of country fixed effects. With the inclusion of country fixed effects the estimates of the contextual effects reflect the effect of variation between city-years within countries. In Model 4 the contextual effect of the local labor market is similar to the effect seen in Model 2, which means that the effect reflects differences between city-years within the same country. On the other hand, in Model 4 the contextual effect of satisfaction with public services is zero, which means that the effect found in Models 2 reflect more differences between countries rather than within countries. In Model 4 the contextual effect of housing is positive but small and statistically significant only at the 10% level.

Regarding the effect of the individual level control variables (see Supplementary Table S4), several variables show a significant effect on financial satisfaction. Gender is one of them, as male respondents tend to be more satisfied with the financial situation of their household. Age has a significant negative association with the dependent variable. We found a negative effect probably because we only considered the young until the age of 35 years, which is the descending part of the U-shaped effect described in the literature (see above). Education is also associated with financial satisfaction as higher education increases the likelihood of being satisfied. Financial satisfaction is also different between occupational groups. Managers are the most satisfied with their financial situation, professionals seem to have the second highest satisfaction, while blue collar workers are more satisfied than their white collar counterparts. Unemployed people are the least satisfied, inactive people and students have lower dissatisfaction. Finally, household composition also plays a role in shaping financial satisfaction. People who are married or live with a partner are significantly more satisfied than those living in single person households and the single parents. In our main models (see Table 2) our city-level control variables GDP *per capita* and city population do not have a statistically significant effect. In case of city population in some of the models we found a significant negative coefficient, which means that in bigger cities, financial satisfaction tends to be slightly lower (see Supplementary Tables S2, S3, S5).

We conducted robustness analysis of our results by including objective indicators of the local labor market and housing market context in our models instead of the subjective indicators used earlier. In case of the labor market context we included the city level unemployment rate, where data have been taken from the OECD metropolitan database (OECD, 2022). In case of the housing market context we included data on rent prices downloaded from Numbeo.com (Numbeo, 2023)^7^. Results of robustness analysis are presented in Table 3. As data was not available for all city-years from these databases the sample used includes a reduced number of city-years, especially in case of rent data where the number of city-years is reduced to 144. Therefore, first we present models where objective indicator is used only in case of the local labor market context (Model 1) and then a model where objective indicators are used for both the local labor and housing market context (Model 2). Our basic results are similar if the unemployment rate is used as a measure of the labor market context instead of the city-level agreement that it is easy to find a job in the city. The effect of the unemployment rate is statistically significant and negative in all models meaning that satisfaction with financial situation tends to be lower in cities or years characterized with a higher unemployment rate. The effect of local housing market context remains small and not significantly different from zero in models when rent prices are used instead of the subjective measures. The effect of satisfaction with public services is unchanged compared to the earlier estimates.

**Table 3:** Effects of objective indicators of local economic context on satisfaction with financial situation among the young (standardized coefficients from multilevel ordered probit models).

	Model (1)	Model (2)	Model (3)	Model (4)
Agree: easy to find job (individual)			0.147^***^	0.182^***^
			(0.000)	(0.000)
Agree: easy to find job (city-year)			0.092^***^	0.097^***^
			(0.000)	(0.000)
Unemployed (indiv., ref. manager)	−0.267^***^	−0.257^***^		
	(0.000)	(0.000)		
Unemployment rate (city-year)	−0.070^**^	−0.104^***^		
	(0.005)	(0.001)		
Agree: easy to find housing (indiv.)	0.153^***^	0.139^***^	0.116^***^	0.138^***^
	(0.000)	(0.000)	(0.000)	(0.000)
Agree: easy to find housing (city-year)	−0.044		0.013	−0.011
	(0.145)		(0.649)	(0.710)
Rent prices		0.046		
		(0.241)		
Satisfaction with services (individual)	0.225^***^	0.216^***^	0.171^***^	0.196^***^
	(0.000)	(0.000)	(0.000)	(0.000)
Satisfaction with services (city-year)	0.079^***^	0.083^**^	0.046^*^	0.067^**^
	(0.001)	(0.006)	(0.028)	(0.002)
Difficulties paying bills: never (ref.)			0.000	
			(.)	
Difficulties paying bills: time to time			−0.267^***^	
			(0.000)	
Difficulties paying bills: always			−0.426^***^	
			(0.000)	
Population	−0.005	−0.004	0.014	−0.030
	(0.828)	(0.886)	(0.495)	(0.160)
GDP *per capita*	0.063^*^	0.033	0.035	0.059^*^
	(0.043)	(0.377)	(0.224)	(0.042)
Educational inequality				0.029
(coeff. of variation)				(0.158)
Individual controls	Yes	Yes	Yes	Yes
Year dummies	Yes	Yes	Yes	Yes
Variance city-year level	0.037	0.039	0.032	0.034
N	27,193	20,742	27,634	28,361
Number of city-years	190	144	205	205
Log-likelihood	−30418.7	−23110.1	−29439.3	−31544.2
LR test	0.000	0.000	0.000	0.000

Standardized beta coefficients; *p*-values in parentheses, ^*^p < 0.05, ^**^p < 0.01, ^***^p < 0.001.

In a second set of robustness checks we included additional control variables in the models. In Model 3 of Table 3 we included a subjective indicator related to the living standards of the household reflecting whether they had difficulties paying bills during the past 12 months. Results show – unsurprisingly – that financial satisfaction is lower among respondents who have difficulties paying bills. The inclusion of this variable in the model however does not change substantively our results regarding the main variables of interest. Model 4 also controlled for an indicator of inequality in the cities. As data on income inequality in the cities was not available, we constructed an indicator of educational inequality from our dataset defined as the coefficient of variation of highest degree of education. The effect of educational inequality is not significantly different from zero and its inclusion in the model does not alter estimates obtained for variables of interest.

In the final step of the analysis we have looked how the effects of our main variables of interest among the young compare to findings in other age groups (the 36–49 and the 50–64 age group). The literature review by Ballas suggests that valuation of characteristics of the city-level context can be different between individuals at different stages of their life-course (Ballas, 2013). The main purpose of this analysis therefore is to see whether the observed effects are specific to the younger age group or whether they can be found in other age groups as well. In this analysis we have pooled data for the age groups and re-estimated our baseline model with including interactions of age group and the individual-level and contextual-level variables of local labor market, local housing market and public services (see detailed results in Supplementary Table S5).

Results of this analysis reveal that there are differences between the age groups in the effect of individual-level perceptions of the city’s economic context and public services. Perception that “it is easy to find a good job in the city” increases financial satisfaction more for those above 35 years of age compared to the young. This is consistent with the finding that among older workers, the duration of unemployment tends to be longer and the chances of finding a job are lower (Axelrad et al., 2018), while the young can more often rely on help from family members in case of unemployment (Gallie and Russell, 1998). Perceiving higher affordability of housing in the city increases financial satisfaction among the young the most, which is consistent with the young being the age group with lowest rates of homeowners and highest rates of renters, who are most constrained by high housing prices (see, e.g., Eurofound, 2023). The important role of housing tenure for moderating the effect of individual perception of housing affordability on financial satisfaction can also be seen from results of our additional analysis which looks at differences across broad country-groups of Europe. As Supplementary Table S6 shows, perceiving affordable housing in the city increases financial satisfaction most in Western and Northern European cities, where the percentage of renters is the highest.

**Figure 2:**
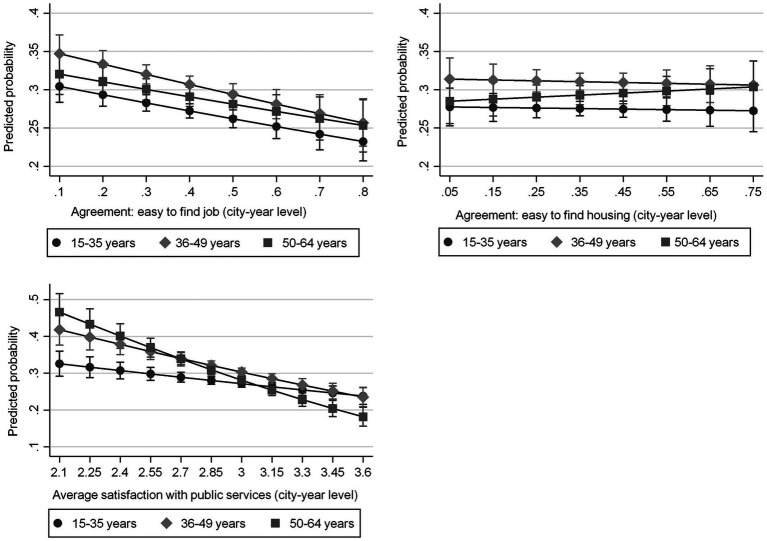
The effect of local economic context and local services on predicted probability of being unsatisfied or very unsatisfied with financial situation (Average adjusted predictions). The figure shows average adjusted predictions of the probability of being unsatisfied or very unsatisfied with the financial situation based on multilevel models described in the Supplementary Table S5.

Regarding the differences between the age groups in the effect of the contextual-level variables our results are summarized in Figure 2. On Figure 2 we show the average adjusted predicted probabilities of being unsatisfied or very unsatisfied with the financial situation of the household at representative values of the variables of interest, separately for different age groups^8^.

In case of the labor market context, a one standard deviation increase in the percentage of those agreeing with “it’s easy to find a job in the city” goes together with a 1.9-point decline in the probability of being unsatisfied with their financial situation among the young (from a baseline of 30.5%). It can also be seen that there is no difference in the effect of labor market context between the young and those between 50 and 64 years of age. The effect among those between 36 and 49 years of age seems to somewhat stronger, but the difference is not statistically significant.

In case of the local housing market there is no significant contextual effect in case of any of the age groups. The probability of being unsatisfied with the financial situation is lower in contexts where general satisfaction with public services is higher in every age group. Among the young a one standard deviation increase in the city-level satisfaction with public services is associated with a 1.7-point decline in the probability of being unsatisfied with the financial situation. It is also clear that the effect of public services is stronger among the other age groups compared to the young, especially in case of those age 50–64 years.

## 5 Discussion

In this study we were interested in the effect of the urban economic context and public services on financial satisfaction among the young. In our multilevel analysis we have found that individual opinions of a better urban labor market, local housing market and local public services are strongly associated with higher satisfaction with finances among the young in European cities. Our main interest in this study however was not the effects of the individual opinions about the local economy and local public services but the contextual effects, meaning the influence of the economic and social environments of the places in which young people live.

Our analysis has confirmed the existence of such contextual effects, especially in case of the urban labor market and local public services. Confirming our first hypothesis we have found that financial satisfaction is higher among the young in cities with a more favorable labor market context. This effect has been found both in models using a subjective indicator of the labor market context (average agreement that it is easy to find a good job in the city) and an objective indicator (the unemployment rate). Moreover, our results show that the urban labor market context is important for well-being of the young even if we look at only variation between city-years within countries. We have thus found similar results using variation between cities to other studies such as Kose and Cinar (2020) and Lee et al. (2023) who analyzed the relation between country-level unemployment rate and financial satisfaction. These results are also similar to findings of studies that have looked at the contextual effect of unemployment on subjective well-being and found that higher unemployment rate in a country leads to lower levels of subjective well-being (eg. Di Tella et al., 2001; Lawless and Lucas, 2011).

To our knowledge this study is the first to analyze the relation between public service quality and financial satisfaction. We have found that satisfaction with public services is positively related to financial satisfaction at the individual level among the young in European cities. Our results have also shown − confirming out third hypothesis − that the young living in cities with better quality local public services (with higher average satisfaction with services) tend to be more satisfied with the financial situation of their household. This finding is in line with the results of earlier studies that have shown the importance of public services on subjective well-being of city residents (eg. Moeinaddini et al., 2020).

Regarding the effect of the urban housing context on financial satisfaction our analysis has shown that financial satisfaction is higher among those young who perceive that housing is affordable in the city. In addition, our results are also in line with the literature suggesting a moderating role of housing tenure in this relationship (see Atalay and Edwards, 2022). We have found that the positive effect of perceived housing affordability on financial satisfaction is the largest in the age-group (the young) and the country-group (Western and Northern Europe) with the highest percentage of renters. Regarding the contextual-level effect of the local housing market we have found only small (and statistically non-significant) effect. To our view this is related to the ambiguous effect of contextual-level housing affordability on financial satisfaction. Unaffordability of housing might have a negative contextual effect as it can lead to a range of social problems with high social costs, which negatively affect well-being of all inhabitants of a city (eg. increased homelessness, decreased health, segregation). In the same time, high housing prices can also be related to attractive aspects of the city environment (such as proximity to jobs, good-quality social services etc.) which increase financial satisfaction among residents of the city. The weak contextual effect of housing found in our analysis is the result of these two opposing forces.

Our results contribute to the literature on the geography of subjective well-being as the effects of the urban context have not been previously studied in case of the young across cities in EU member states. Our analysis has confirmed the existence of contextual effects in case of the urban labor market and local public services. On the other hand, we have found no evidence that context matters more in case of the young compared to other age groups. The importance of the labor market and housing market context proved to be similar for all age groups while in case of public services, the contextual effect was smaller among the young compared to other age groups.

Our study provides new insights in the analysis of contextual effects on well-being among the urban young in Europe. There are however certain limitations of the study that have to be taken into account. Our study is focusing on urban areas but the dataset used offers a limited coverage of cities within each country. In addition, while the survey includes a rich set of items regarding subjective well-being and satisfaction with aspects of life in the cities, detailed measurement of some of the relevant control variables (eg. household income or housing tenure) is missing from the survey. Another limitation is that the survey follows a repeated cross-sectional design, which means that a different sample of respondents were asked in each wave. Such design does not allow to study the effect of changes in the city-level context on changes in individual financial satisfaction, which inhibits strong causal claims at the individual level. Despite these limitations, to our knowledge this is the only cross-country dataset to study quality of life in cities of Europe.

## Data availability statement

Publicly available datasets were analyzed in this study. This data can be found here: GESIS Data Archive, Cologne, ZA6641 Data file Version 1.0.0, https://doi.org/10.4232/1.12516; GESIS Data Archive, Cologne, ZA5885 Data file Version 1.1.0, https://doi.org/10.4232/1.12910; European Commission (2019). Quality of Life in European Cities https://ec.europa.eu/regional_policy/information-sources/maps/quality-of-life_en.

## Author contributions

MM: original idea, study design, data analysis, interpretation of results, and manuscript drafting. ÁC: data analysis, interpretation of results, and manuscript drafting. All authors contributed to the article and approved the submitted version.

## Footnotes

^1^The authors compared the impact of satisfaction with financial situation, job, accommodation, commuting time, time use, personal relations, green areas and environment, and they found that financial satisfaction has the strongest association with overall life satisfaction.

^2^Kaiser and Vendrik (2019) however argue, that while Easterlin-paradox is more or less robust for economically developed, Western and Northern European countries, we get less clear evidence of Easterlin’s main points for some Southern and Eastern European countries.

^3^The authors argue that the effect of an increase in housing prices can be positive for homeowners who have taken out a mortgage as the increase in housing wealth increases the value of the home as a collateral (Atalay and Edwards, 2022).

^4^In 2019, the survey also included data from Albania, North Macedonia, Montenegro, and Serbia, but these data are not used in the analysis.

^5^Multilevel models, where the variables of interest were included in the model one-by-one together with macro-level controls (city population), individual level controls and year dummies show similar results. Results are shown in the Supplementary Table S3.

^6^In models where public services were included separately results show that contextual level satisfaction with education services, health care services and public transport were all positively related to financial satisfaction among the young.

^7^We used the data series on rent per month for one-bedroom apartment outside the city center (Numbeo, 2023).

^8^Predicted probabilities were calculated at representative values of the variables of interest while all other variables were kept at their observed values for every case in the sample. Predicted probabilities were then averaged over all cases.
