# Supplementary material for: The effect of the local economic context and local public services on financial satisfaction among youth in European cities
Source: Front Sociol. 2024 Mar 8;9:1207807. doi: 10.3389/fsoc.2024.1207807 (PMC10957626; doi:10.3389/fsoc.2024.1207807)
Supplement: Supplementary file 1 [file Data_Sheet_1.docx]

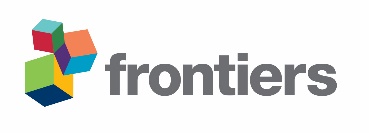


Supplementary Material

The effect of the local economic context and local public services on financial satisfaction among youth in European cities

Márton Medgyesi*, Ábel Csathó

*** Correspondence:** Corresponding Author: [medgyesi@tarki.hu](mailto:medgyesi@tarki.hu)

**Supplementary Figure S1** Distribution of satisfaction with financial situation in the 15-35 age group (%, pooled sample over years 2012, 2015, 2019)


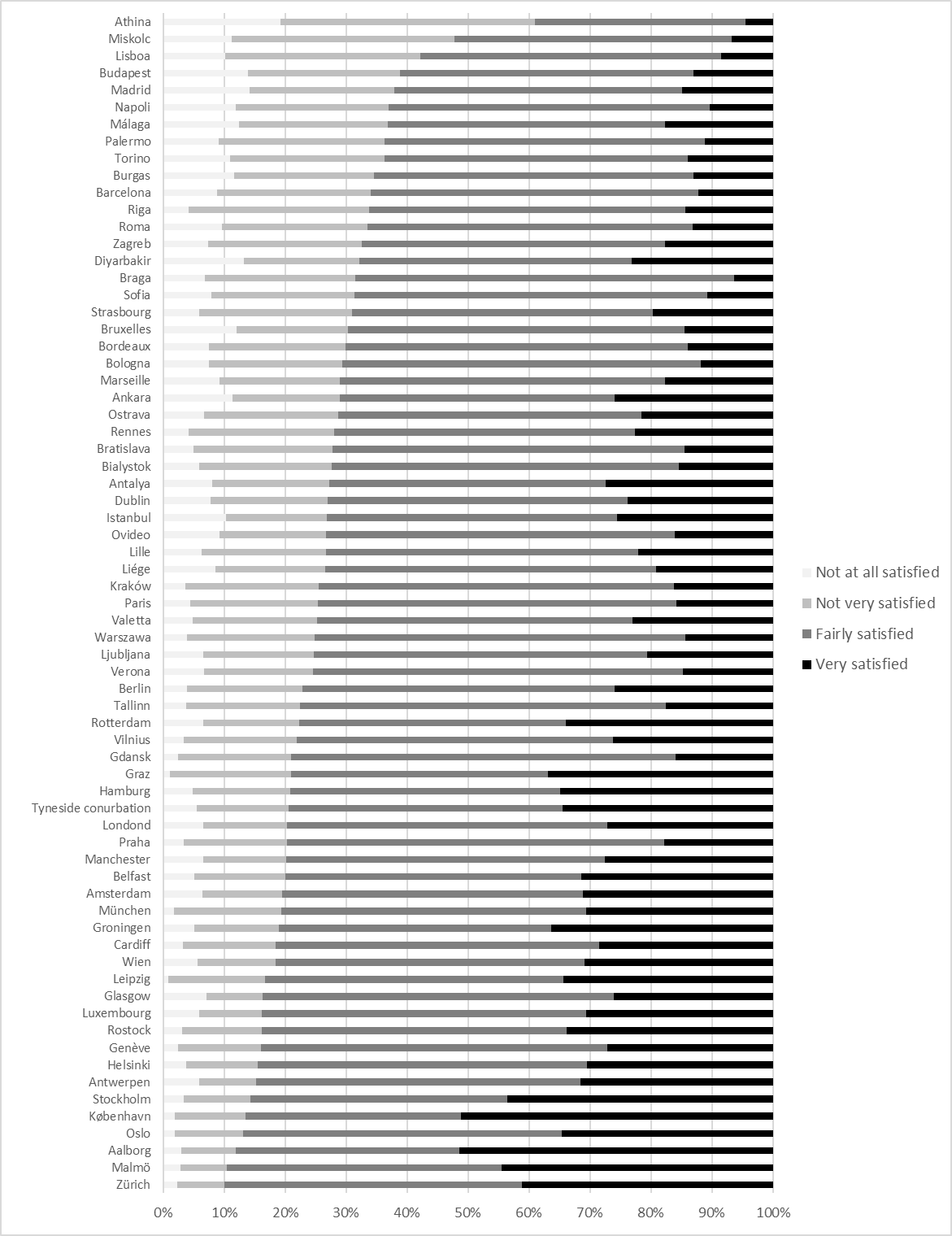


Note: Descriptive statistics were calculated on the weighted database. Weights are based on age and gender.

**Supplementary Table S1** Descriptives of independent variables in the regression analysis

|  | N | Mean | Standard deviation | Mini-mum | Maxi-mum |
| --- | --- | --- | --- | --- | --- |
| Female | 28130 | 0.499 | 0.500 | 0 | 1 |
| Age | 28130 | 26.625 | 5.663 | 15 | 35 |
| Education: less than upper secondary | 28130 | 0.068 | 0.252 | 0 | 1 |
| Education: upper secondary | 28130 | 0.445 | 0.497 | 0 | 1 |
| Education: tertiary | 28130 | 0.487 | 0.500 | 0 | 1 |
| Occupation: manager | 28130 | 0.075 | 0.264 | 0 | 1 |
| Occupation: professional | 28130 | 0.107 | 0.309 | 0 | 1 |
| Occupation: white collar | 28130 | 0.346 | 0.476 | 0 | 1 |
| Occupation: blue collar | 28130 | 0.111 | 0.314 | 0 | 1 |
| Occupation: unemployed | 28130 | 0.094 | 0.291 | 0 | 1 |
| Occupation: student | 28130 | 0.221 | 0.415 | 0 | 1 |
| Occupation: inactive | 28130 | 0.047 | 0.211 | 0 | 1 |
| Household: single person household | 28130 | 0.154 | 0.361 | 0 | 1 |
| Household: couple no children in hhd. | 28130 | 0.208 | 0.406 | 0 | 1 |
| Household: single parent | 28130 | 0.081 | 0.272 | 0 | 1 |
| Household: couple w children in hhd. | 28130 | 0.416 | 0.493 | 0 | 1 |
| Household: other | 28130 | 0.139 | 0.346 | 0 | 1 |
| Agree easy to find job (individual) | 28130 | 0.464 | 0.499 | 0 | 1 |
| Agree easy to find housing (individual) | 28130 | 0.362 | 0.481 | 0 | 1 |
| Satisfaction w. public services (indiv.) ()(indiv) | 28130 | 2.967 | 0.648 | 1 | 4 |
| *City-year level variables:* |  |  |  |  |  |
| Population | 28130 | 2.251 | 2.827 | 0.248 | 13.320 |
| GDP per capita | 28130 | 4.524 | 1.736 | 1.095 | 10.773 |
| % agree easy to find job | 28130 | 0.399 | 0.184 | 0.015 | 0.811 |
| Unemployment rate (%) | 25835 | 9.329 | 5.619 | 1.400 | 34.400 |
| % agree easy to find housing | 28130 | 0.361 | 0.196 | 0.029 | 0.776 |
| Satisfaction with education | 28130 | 0.296 | 0.127 | 0.062 | 0.658 |
| Satisfaction with health care | 28130 | 0.305 | 0.155 | 0.035 | 0.716 |
| Satisfaction with public transport | 28130 | 0.321 | 0.133 | 0.015 | 0.835 |
| Satisfaction with public services | 28130 | 2.978 | 0.298 | 2.098 | 3.650 |

Note: Descriptive statistics were calculated on the weighted database. Weights are based on age and gender.

**Supplementary Table S2** City-year level variance from intercept-only model and model with individual controls (dependent variable: financial satisfaction)

|  | (1) | (2) | (3) |
| --- | --- | --- | --- |
|  | 15-35  age group | 36-49  age group | 50-64  age group |
| Null models with only random intercept | | | |
| Variance city-year level | 0.092 | 0.146 | 0.199 |
| N | 28130 | 24141 | 24181 |
| Number of city-years | 205 | 205 | 205 |
| Log-likelihood | -32603.6 | -28241.9 | -28136.6 |
| LR test | 0.000 | 0.000 | 0.000 |
| Null models with individual controls | | | |
| Variance city-year level | 0.085 | 0.135 | 0.191 |
| N | 28130 | 24141 | 24181 |
| Number of city-years | 205 | 205 | 205 |
| Log-likelihood | -32057.5 | -27503.2 | -27447.6 |
| LR test | 0.000 | 0.000 | 0.000 |

**Supplementary Table S3** Multilevel models with contextual variables introduced separately (dependent var.: financial satisfaction)

|  | (1) | (2) | (3) | (4) | (5) | (6) | (7) |
| --- | --- | --- | --- | --- | --- | --- | --- |
| Population | -0.082^**^ | -0.072^**^ | -0.077^**^ | -0.016 | -0.055^*^ | -0.070^**^ | -0.025 |
|  | (0.007) | (0.006) | (0.008) | (0.006) | (0.006) | (0.007) | (0.006) |
| GDP per capita | 0.174^***^ | 0.069^**^ | 0.176^***^ | 0.103^***^ | 0.091^***^ | 0.124^***^ | 0.075^**^ |
|  | (0.012) | (0.012) | (0.017) | (0.010) | (0.010) | (0.011) | (0.011) |
| Agree: easy to find job (individual) |  | 0.218^***^ |  |  |  |  |  |
|  |  | (0.014) |  |  |  |  |  |
| Agree: easy to find job (city-year level) |  | 0.118^***^ |  |  |  |  |  |
|  |  | (0.108) |  |  |  |  |  |
| Agree: easy to find housing (individual) |  |  | 0.177^***^ |  |  |  |  |
|  |  |  | (0.015) |  |  |  |  |
| Agree: easy to find housing (city-year l.) |  |  | -0.066 |  |  |  |  |
|  |  |  | (0.148) |  |  |  |  |
| Satisfied w. education services (individ.) |  |  |  | 0.125^***^ |  |  |  |
|  |  |  |  | (0.015) |  |  |  |
| Satisfied w. education services (city-year l) |  |  |  | 0.157^***^ |  |  |  |
|  |  |  |  | (0.138) |  |  |  |
| Satisfied w. health care (individual) |  |  |  |  | 0.139^***^ |  |  |
|  |  |  |  |  | (0.015) |  |  |
| Satisfied w. health care (city-year level) |  |  |  |  | 0.154^***^ |  |  |
|  |  |  |  |  | (0.110) |  |  |
| Satisfied w. public transport (individual) |  |  |  |  |  | 0.106^***^ |  |
|  |  |  |  |  |  | (0.015) |  |
| Satisfied w. public transport (city-year l.) |  |  |  |  |  | 0.131^***^ |  |
|  |  |  |  |  |  | (0.131) |  |
| Average satisfaction w. services (individ.) |  |  |  |  |  |  | 0.237^***^ |
|  |  |  |  |  |  |  | (0.011) |
| Average satisfaction w. services (city-year) |  |  |  |  |  |  | 0.110^***^ |
|  |  |  |  |  |  |  | (0.062) |
| Variance city-year level | 0.067 | 0.047 | 0.064 | 0.042 | 0.043 | 0.052 | 0.045 |
| N | 31195 | 29333 | 29697 | 28506 | 30438 | 29696 | 31173 |
| Number of city-years | 205 | 205 | 205 | 205 | 205 | 205 | 205 |
| Log-likelihood | -35408.0 | -33023.9 | -33532.3 | -32287.1 | -34383.5 | -33593.8 | -34945.4 |
| LR test | 0.000 | 0.000 | 0.000 | 0.000 | 0.000 | 0.000 | 0.000 |

Note: Standardized beta coefficients; Standard errors in parentheses ^*^ *p* < 0.05 ^**^ *p* < 0.01 ^***^ *p* < 0.00. All models include individual controls and year dummies.

**Supplementary Table S4** Effects of individual level control variables (dependent var.: financial satisfaction)

|  | (1) | (2) | (3) | (4) |
| --- | --- | --- | --- | --- |
| Female | -0.022^**^ | -0.016^*^ | -0.021^*^ | -0.015 |
|  | (0.006) | (0.049) | (0.010) | (0.074) |
| Age | -0.157^***^ | -0.150^***^ | -0.154^***^ | -0.147^***^ |
|  | (0.000) | (0.000) | (0.000) | (0.000) |
| Less than upper secondary | ref. | ref. | ref. | ref. |
|  |  |  |  |  |
| Upper secondary | 0.039^*^ | 0.044^**^ | 0.039^*^ | 0.043^*^ |
|  | (0.020) | (0.010) | (0.020) | (0.011) |
| Tertiary education | 0.092^***^ | 0.095^***^ | 0.090^***^ | 0.092^***^ |
|  | (0.000) | (0.000) | (0.000) | (0.000) |
| Manager | ref. | ref. | ref. | ref. |
|  |  |  |  |  |
| Professional | -0.071^***^ | -0.074^***^ | -0.073^***^ | -0.076^***^ |
|  | (0.000) | (0.000) | (0.000) | (0.000) |
| White collar | -0.188^***^ | -0.179^***^ | -0.190^***^ | -0.181^***^ |
|  | (0.000) | (0.000) | (0.000) | (0.000) |
| Blue collar | -0.138^***^ | -0.131^***^ | -0.143^***^ | -0.137^***^ |
|  | (0.000) | (0.000) | (0.000) | (0.000) |
| Unemployed | -0.268^***^ | -0.244^***^ | -0.270^***^ | -0.247^***^ |
|  | (0.000) | (0.000) | (0.000) | (0.000) |
| Student | -0.142^***^ | -0.139^***^ | -0.145^***^ | -0.142^***^ |
|  | (0.000) | (0.000) | (0.000) | (0.000) |
| Inactive | -0.131^***^ | -0.126^***^ | -0.132^***^ | -0.128^***^ |
|  | (0.000) | (0.000) | (0.000) | (0.000) |
| Single person household | ref. | ref. | ref. | ref. |
|  |  |  |  |  |
| Household: couple no children in hhd | 0.030^**^ | 0.036^**^ | 0.031^**^ | 0.037^***^ |
|  | (0.006) | (0.001) | (0.005) | (0.001) |
| Household: single parent | -0.031^**^ | -0.024^*^ | -0.028^**^ | -0.021^*^ |
|  | (0.001) | (0.012) | (0.003) | (0.029) |
| Household: couple w children in hhd | 0.037^**^ | 0.045^***^ | 0.039^**^ | 0.047^***^ |
|  | (0.003) | (0.000) | (0.001) | (0.000) |
| Household: Other | -0.002 | 0.002 | -0.003 | 0.000 |
|  | (0.875) | (0.882) | (0.756) | (0.973) |
| Macro-level variables | Yes | Yes | Yes | Yes |
| Year dummies | Yes | Yes | Yes | Yes |
| Country dummies | No | No | Yes | Yes |
| Variance city-year level | 0.032 | 0.034 | 0.002 | 0.002 |
| N | 28130 | 28130 | 28130 | 28130 |
| Number of city-years | 205 | 205 | 205 | 205 |
| Log-likelihood | -31974.5 | -31256.2 | -31840.8 | -31123.2 |
| LR test | 0.000 | 0.000 | 0.011 | 0.004 |

Note: *p*-values in parentheses ^*^ *p* < 0.05, ^**^ *p* < 0.01, ^***^ *p* < 0.001

**Supplementary Table S5** Pooled models across age groups with interactions of age and variables of interest (dependent variable: financial satisfaction)

|  | standardized coefficients |  | p-values |
| --- | --- | --- | --- |
| 15-35 years old | ref. |  | (.) |
| 36-49 years old | -0.646 | * | (0.017) |
| 50-64 years old | -1.222 | *** | (0.000) |
| Agreement: easy to find job (individual) | 0.290 | *** | (0.000) |
| 15-35 years old # Agreement: easy to find job | ref. |  | (.) |
| 36-49 years old # Agreement: easy to find job | 0.046 | * | (0.033) |
| 50-64 years old # Agreement: easy to find job | 0.054 | * | (0.016) |
| Agreement: easy to find housing (individual) | 0.250 | *** | (0.000) |
| 15-35 years old # Agreement: easy to find housing | ref. |  | (.) |
| 36-49 years old # Agreement: easy to find housing | -0.044 | * | (0.049) |
| 50-64 years old # Agreement: easy to find housing | -0.068 | ** | (0.003) |
| Satisfaction with services (individual) | 0.257 | *** | (0.000) |
| 15-35 years old # Satisfaction with services | ref. |  | (.) |
| 36-49 years old # Satisfaction with services | 0.021 |  | (0.215) |
| 50-64 years old # Satisfaction with services | 0.023 |  | (0.174) |
| *City-year level variables and cross-level interactions:* |  |  |  |
| Agreement: easy to find job (city-year level) | 0.319 | ** | (0.008) |
| 15-35 years old # Agreement: easy to find job | ref. |  | (.) |
| 36-49 years old # Agreement: easy to find job | 0.020 |  | (0.906) |
| 50-64 years old # Agreement: easy to find job | -0.083 |  | (0.620) |
| Agreement: easy to find housing (city-year level) | 0.028 |  | (0.811) |
| 15-35 years old # Agreement: easy to find housing | ref. |  | (.) |
| 36-49 years old # Agreement: easy to find housing | 0.037 |  | (0.798) |
| 50-64 years old # Agreement: easy to find housing | -0.055 |  | (0.709) |
| Satisfaction with services (city-year level) | 0.200 | ** | (0.003) |
| 15-35 years old # Satisfaction with services | ref. |  | (.) |
| 36-49 years old # Satisfaction with services | 0.154 |  | (0.102) |
| 50-64 years old # Satisfaction with services | 0.395 | *** | (0.000) |
| Contextual controls | Yes |  | Yes |
| Individual controls | Yes |  | Yes |
| Year dummies | Yes |  | Yes |
| N | 76452 |  |  |
| Number of city-years | 205 |  |  |
| Log-likelihood | -85001.9 |  |  |

Note: *p*-values in parentheses ^*^ *p* < 0.05, ^**^ *p* < 0.01, ^***^ *p* < 0.001

**Supplementary Table S6** Models with interactions of regions and variables of interest (dependent variable: financial satisfaction)

|  | standardized coefficients |  | p-values |
| --- | --- | --- | --- |
| Eastern EU | ref. |  | (.) |
| West&North EU | 1.114 |  | (0.054) |
| Southern EU | 1.146 | * | (0.023) |
| Agreement: easy to find a good job (individual) | 0.285 | *** | (0.000) |
| Eastern EU # Agreement: easy to find job | ref. |  | (.) |
| West&North EU # Agreement: easy to find job | 0.009 |  | (0.797) |
| Southern EU # Agreement: easy to find job | 0.040 |  | (0.339) |
| Agreement: easy to find housing (individual) | 0.192 | *** | (0.000) |
| Eastern EU # Agreement: easy to find housing | ref. |  | (.) |
| West&North EU # Agreement: easy to find housing | 0.086 | * | (0.022) |
| Southern EU # Agreement: easy to find housing | 0.051 |  | (0.193) |
| Satisfaction with services (individual) | 0.269 | *** | (0.000) |
| Eastern EU # Satisfaction with services | ref. |  | (.) |
| West&North EU # Satisfaction with services | -0.026 |  | (0.377) |
| Southern EU # Satisfaction with services | -0.016 |  | (0.603) |
| *City-year level variables and cross-level interactions:* |  |  |  |
| Agreement: easy to find job (city-year level) | 0.203 |  | (0.280) |
| Eastern EU # Agreement: easy to find job | ref. |  | (.) |
| West&North EU # Agreement: easy to find job | 0.668 | ** | (0.005) |
| Southern EU # Agreement: easy to find job | 0.598 | * | (0.023) |
| Agreement: easy to find housing (city-year level) | -0.016 |  | (0.947) |
| Eastern EU # Agreement: easy to find housing | ref. |  | (.) |
| West&North EU # Agreement: easy to find housing | 0.151 |  | (0.552) |
| Southern EU # Agreement: easy to find housing | -0.116 |  | (0.681) |
| Satisfaction with services (city-year level) | 0.260 |  | (0.052) |
| Eastern EU # Satisfaction with services | ref. |  | (.) |
| West&North EU # Satisfaction with services | -0.400 | * | (0.036) |
| Southern EU # Satisfaction with services | -0.398 | * | (0.023) |
| Contextual controls | Yes |  |  |
| Individual controls | Yes |  |  |
| Year dummies | Yes |  |  |
| N | 28130 |  |  |
| Number of city-years | 205 |  |  |
| Log-likelihood | -31327.7 |  |  |

Note: *p*-values in parentheses ^*^ *p* < 0.05, ^**^ *p* < 0.01, ^***^ *p* < 0.001
